# Evaluation of autoimmune and inflammatory markers in bilateral sudden hearing loss

**DOI:** 10.1007/s00405-025-09414-9

**Published:** 2025-04-25

**Authors:** Aynur Aliyeva, Elif Sari

**Affiliations:** 1 Division of Otorhinolaryngology-Head and Neck Surgery, The Catholic University St.Mary Hospital Medical Center, Seoul, South Korea; 2 The Neuroscience Doctoral Program, Yeditepe University, Istanbul, Turkey; 3 Department of Otorhinolaryngology-Head and Neck Surgery, Istanbul Aydın University VM Medikal Park Florya Hospital, Istanbul, Turkey

**Keywords:** Autoimmune markers, Inflammatory markers, Bilateral sudden hearing loss, Treatment outcomes, Hearing recovery prognosis

## Abstract

**Background:**

Bilateral sudden hearing loss (BSHL) is a rare otologic emergency, representing 1–2% of all sudden hearing loss cases. Autoimmune processes are considered one of the key contributors to BSHL, with immune responses targeting inner ear structures. The aim of our study is to identify the role of autoimmune and inflammatory markers in patients with BSHL and assess their prognostic significance.

**Materials and methods:**

This retrospective study reviewed 30 BSHL patients out of 420 sudden hearing loss cases. Data including audiometric results, medical histories, autoimmune and inflammatory markers (ANA, RF, ESR, CRP, ACA, ANCA), and autoimmune diseases such as Ankylosing Spondylitis (AS), Hypothyroidism (HT), Rheumatoid Arthritis (RA), and Systemic Lupus Erythematosus (SLE) were analyzed. Audiometric recovery was assessed using Siegel’s criteria, and the presence of autoimmune markers was evaluated for their impact on clinical outcomes and recovery.

**Results:**

Autoimmune markers were detected in 60% of the patients with BSHL, with elevated levels of ANA, RF, ESR, and CRP observed most frequently. Patients who tested positive for autoimmune markers demonstrated significantly lower recovery rates compared to those without marker positivity. Specifically, 13% of patients with positive markers showed partial hearing recovery, while 100% of patients without markers exhibited partial recovery. The average hearing improvement across all frequencies was around 21.5 dB. These results suggest that autoimmune marker positivity is associated with poorer clinical outcomes and lower recovery rates following standard corticosteroid treatment.

**Conclusion:**

Our study demonstrates that autoimmune and inflammatory markers play a significant role in the prognosis of BSHL. Patients with positive autoimmune markers exhibit poorer recovery rates, indicating the potential importance of these markers in predicting treatment outcomes for BSHL.

## Highlights

Autoimmune Marker Detection: Autoimmune markers were detected in 60% of BSHL patients, with ANA, RF, ESR, and CRP being the most frequently elevated markers.

Reduced Recovery in Marker-Positive Patients: Patients with positive autoimmune markers exhibited significantly lower recovery rates, with only 13% showing partial hearing recovery compared to 100% without markers.

Hearing Improvement: The average hearing improvement across all frequencies in the study was approximately 21.5 dB, indicating the impact of treatment on overall auditory function.

Prognostic Significance: Autoimmune and inflammatory markers were associated with poorer clinical outcomes and slower recovery following standard corticosteroid therapy.

Gender Differences in Autoimmune Marker Distribution: The study found notable differences in autoimmune marker distribution between genders, particularly in RF and CRP levels.

## Introduction

Sudden Hearing Loss (SHL) is a sensorineural hearing loss that develops within 72 h, resulting in a loss greater than 30 dB across at least three consecutive frequencies [1]. SHL is considered an otologic emergency, with an incidence of 5–20 per 100,000 individuals. Bilateral SHL (BSHL) is rarer, representing only 1–2% of cases, with no gender or ear laterality preference [2, 3]. One of the most common causes of BSHL is thought to be autoimmune processes. The autoimmune theory postulates a localized immune response within the inner ear, with the endolymphatic sac identified as a key immunologically active region [4]. This theory is supported by findings such as elevated expression of significant histocompatibility complex class II genes, circulating immune complexes, increased levels of nonspecific and specific inner ear autoantibodies (e.g., anti-HSP70), and abnormalities in T lymphocyte subpopulations.

The primary objective of this study is to assess the presence of autoimmune and inflammatory markers in patients with bilateral SHL, alongside the clinical course, treatment regimens, radiological findings, and laboratory data, to determine the prognosis of bilateral autoimmune-related SHL. Additionally, the study aims to explore the prognostic significance of autoimmune markers in guiding treatment outcomes. Given the limited research available in this area, this study intends to contribute valuable insights to the literature on the role of autoimmunity in bilateral SHL. This study hypothesizes that autoimmune mechanisms play a crucial role in developing bilateral SHL and that these cases can be linked to specific autoimmune markers. By evaluating clinical, radiological, and laboratory findings, we aim to determine how autoimmune markers influence the prognosis and treatment response in patients with bilateral SHL.

## Materials and methods

This retrospective study was conducted on patients admitted to the Ear, Nose, and Throat (ENT) of Istanbul Aydin University Florya Hospital from January 2021 to October 2024 will be included in the study, who were diagnosed with sudden hearing loss (SHL). Four hundred twenty patients were screened, and 30 cases of bilateral sudden hearing loss (BSHL) were identified. These patients’ medical histories, audiometric data, radiological findings, treatment regimens, clinical progress, and laboratory results were analyzed.

Patients with bilateral sudden hearing loss (SHL), defined as a sensorineural loss of more than 30 dB across at least three consecutive frequencies within 72 h, were included in the study. Autoimmune activity was assessed through tests for Antinuclear Antibody (ANA), Rheumatoid Factor (RF), Erythrocyte Sedimentation Rate (ESR), C-Reactive Protein (CRP), Anticardiolipin Antibody (ACA), and Antineutrophil Cytoplasmic Antibodies (ANCA). Exclusion criteria included patients with known causes of hearing loss, prior ear surgeries, those under 18 or over 65, pregnant or breastfeeding women, and individuals with pre-existing autoimmune diseases.

### Study design

This retrospective clinical study focused on patients diagnosed with bilateral sudden hearing loss (SHL). Data were collected from the records of 30 bilateral SHL cases out of 420 SHL patients. The information analyzed included medical histories, audiometric results, radiological imaging, treatment protocols, clinical progression, and laboratory results. Autoimmune and systemic conditions like Ankylosing Spondylitis (AS), Hypothyroidism (HT), Rheumatoid Arthritis (RA), and Systemic Lupus Erythematosus (SLE) were also noted. Laboratory tests included routine blood work and specialized autoimmune markers (ANA, RF, ESR, CRP, ACA, and ANCA). The primary outcome was to evaluate autoimmune and inflammatory markers and clinical and radiological data to assess the prognosis of autoimmune-related bilateral SHL.

The data were statistically analyzed to assess the presence of autoimmune markers in patients with bilateral SHL and their impact on clinical outcomes. The relationship between autoimmune markers and prognosis was evaluated. Pure tone and speech audiometry were conducted using calibrated clinical audiometers in a soundproof cabin, measuring hearing thresholds from 250 Hz to 8 kHz. Pure tone averages (PTA) were calculated as the mean of thresholds at 500, 1000, 2000, and 4000 Hz.

According to the American Speech–Language–Hearing Association (ASHA), mild hearing loss is defined as the inability to hear sounds between 26 and 39 dB, moderate loss between 40 and 54 dB, moderately severe between 55 and 69 dB, severe between 70 and 89 dB, and profound above 90 dB. Hearing thresholds at various frequencies before and after treatment were recorded. Siegel’s criteria assessed recovery, defining complete recovery as a difference in PTA of up to 25 dB, partial recovery as 26–44 dB, slight improvement as 45–74 dB, and no improvement over 75 dB [5–8].

This study was designed at the Istanbul Aydin University VM Medical Park Florya Hospital. The Istanbul Aydin University clinical research ethics committee approved it (approval *N* = 126/2024 and date 11/12/2024).

### Statistical and analytical methods

Statistical analyses were performed using IBM Statistical Package for the Social Sciences (SPSS) version 25 (SPSS Inc.; Chicago, IL, USA). The normal distribution of variables was assessed using visual methods (histograms and probability plots) and analytical tests (Kolmogorov-Smirnov/Shapiro-Wilk test). Descriptive and analytical statistics were reported as counts, percentages, means, standard deviations, medians, and minimum-maximum values, depending on the normality of distribution. Comparisons between dependent groups were made using paired t-tests or Mann-Whitney U tests, and comparisons between multiple groups were made using ANOVA or Kruskal-Wallis tests. A Type 1 error level of 0.05 was used to determine statistical significance.

## Results

The average age of the 30 patients was evaluated; the average age was 51.53 ± 12.65 years. The youngest patient was 25 years old, and the oldest was 65 years old. Among the 30 patients, 20 (66.67%) were female, and 10 were male (33.33%), indicating a higher prevalence of females in the study population. Autoimmune and inflammatory markers were observed in varying frequencies among the patients: ANA and RF were elevated in 8 patients each (26.67%), ESR and CRP were elevated in 9 patients each (30.0%), ANCA was detected in 3 patients (10.0%) (Table 1). Here is a breakdown of the combinations of autoimmune and inflammatory markers observed:

**Table 1:** Distribution of autoimmune markers and autoimmune diseases in BSHL patients

Autoimmune Markers			Autoimmune Diseases		
Name	**Number**	**Percentage**	Name	**Number**	**Percentage**
ANA	8	26.67%	AS	5	16.67%
RF	8	26.67%	HT	4	13.33%
ESR	9	30%	RA	8	26.67%
CRP	9	30%	SLE	9	30%
ACA	4	13.3%			
ANCA	3	10%			

Antinuclear Antibody (ANA), Rheumatoid Factor (RF), Erythrocyte Sedimentation Rate (ESR), C-reactive protein (CRP), Antineutrophil Cytoplasmic Antibodies (ANCA), Anticardiolipin Antibody (ACA). Additionally, Ankylosing Spondylitis (AS), Hypothyroidism (HT), Rheumatoid Arthritis (RA), Systemic Lupus Erythematosus (SLE)

- 1 marker: This was seen in 11 patients, where each had only one of the following markers: ANA, RF, ESR, or CRP.
- 2 markers together: In 10 patients, two markers were observed together. The most common combinations included ANA and RF, ESR and CRP, or combinations like RF and ESR.
- 3 markers together: In 2 patients, three markers were observed together. These included combinations like ANA, RF, and ESR or RF, ESR, and CRP.
- 4 markers together: In 1 patient, 4 markers were observed together, which included ANA, RF, ESR, and CRP.

Notably, no autoimmune or inflammatory markers were detected in 6 out of 30 patients (20%), while the remaining 24 (80%) exhibited at least one positive marker. The prevalence of diseases in the patient population was as follows: Ankylosing Spondylitis (AS) was observed in 5 patients (16.67%), Hypothyroidism (HT) in 4 patients (13.33%), Rheumatoid Arthritis (RA) in 8 patients (26.67%), and Systemic Lupus Erythematosus (SLE) in 9 patients (30.0%).

Among female patients, 5 out of 20 (25%) had elevated ANA levels, 3 patients (15%) had elevated RF levels, 6 patients (30%) had elevated ESR levels, 8 patients (40%) had elevated CRP levels, 3 patients (15%) had elevated ACA levels, and 2 patients (10%) had elevated ANCA levels. In terms of diseases, Ankylosing Spondylitis (AS) was observed in 3 female patients (15%), Hypothyroidism in 2 female patients (10%), Rheumatoid Arthritis (RA) in 5 female patients (25%), and Systemic Lupus Erythematosus (SLE) in 6 female patients (30%).

Among male patients, 3 out of 10 (30%) had elevated ANA levels, 5 patients (50%) had elevated RF levels, 3 patients (30%) had elevated ESR levels, 1 patient (10%) had elevated CRP levels, 1 patient (10%) had elevated ACA levels, and 1 patient (10%) had elevated ANCA levels. Regarding diseases, Ankylosing Spondylitis (AS) was observed in 2 male patients (20%), Hypothyroidism in 2 male patients (20%), Rheumatoid Arthritis (RA) in 3 male patients (30%), and Systemic Lupus Erythematosus (SLE) in 3 male patients (30%).

This shows a notable difference in the distribution of some markers and diseases between genders, particularly in RF and CRP, where males had a higher frequency of RF with 5 out of 10 patients (50%), compared to 3 out of 20 female patients (15%). In contrast, females had a higher frequency of CRP with 8 out of 20 patients (40%), compared to 1 out of 10 male patients (10%).

All patients received the same treatment protocol, which consisted of systemic corticosteroid therapy with intravenous methylprednisolone at a dose of 1 mg/kg/day for 7 days, followed by a tapering dose of oral steroids over the subsequent week, along with proton pump inhibitor (PPI) prophylaxis to prevent gastrointestinal side effects.; their radiological evaluations were unremarkable. Hemogram and biochemical values showed no statistically significant differences compared to unilateral SHL patients.

After a follow-up period averaging 18.17 ± 5.28 months (range: 11–29 months), it was found that patients who tested positive for autoimmune and inflammatory markers showed only slight clinical improvement. The lack of substantial recovery among these patients highlights the impact of autoimmunity on their prognosis.

### Left ear audiometric parameters

Left ear audiometric parameters revealed that 20 out of 30 patients (66.67%) initially had severe hearing loss, while the remaining 10 patients (33.33%) presented with profound hearing loss. On average, treatment resulted in a hearing improvement of around 22.33 dB across frequencies. The pure tone average (PTA) improved from 87.50 ± 9.38 dB before treatment to 65.17 ± 12.19 dB afterward. Based on Siegel’s criteria, 22 out of 30 patients (73.34%) showed improvement, with 20 patients (66.67%) experiencing mild improvement and 2 patients (6.67%) showing partial improvement, while 8 patients (26.67%) had no improvement (Table 2).

**Table 2:** Audiometric outcomes and Frequency-Specific hearing gains before and after treatment

Ear (*n*)	Pretreatment PTA (dB),	Post-treatment PTA (dB)	Average Gain (dB)	Mild Improvement	Partial Improvement	No Improvement
**Left Ear (30)**	87.50 ± 9.38,	65.17 ± 12.19	22.33	20 (66.7%)	2(6.67%)	8 (26.67%)
**Right Ear (30)**	87.58 ± 16.39	67.53 ± 21.40	20.06	19 (63.33%)	2 (6.67%)	9 (30%)
**Total (60)**	**N/A**	**N/A**	**N/A**	39 (65%),	4 (6.67%)	17 (28.33%)
**Frequency-Specific Pre- and Post-Treatment Hearing Thresholds in Left**, **Right**						
**Left ear**						
	**PRE250**	**PRE500**	**PRE1000**	**PRE2000**	**PRE4000**	**PREPTA**
**Mean**	80	80.5	86.7	91.2	91.7	87.5
**SD**	**12.11**	**13.54**	**11.32**	**10.56**	**11.55**	**9.38**
	**POST250**	**POST500**	**POST1000**	**POST2000**	**POST4000**	**POSTPTA**
**Mean**	58	59	63.5	68.5	69.7	65.2
**SD**	**11.72**	**12.28**	**15.76**	**15.21**	**16.76**	**12.19**
	**250GAIN**	**500GAIN**	**1000GAIN**	**2000GAIN**	**4000GAIN**	
**Mean**	22	21.5	23.2	22.7	22	
**SD**	13.2	12.3	18	16.1	14.2	
**t test**	8.11E-10	1.25E-08	8.72E-09	4.57E-09	9.22E-08	
**p value**	*p* < 0.05	*p* < 0.05	*p* < 0.05	*p* < 0.05	*p* < 0.05	
**Righ ear**						
**Right ear**	**PRE250**	**PRE500**	**PRE1000**	**PRE2000**	**PRE4000**	**PREPTA**
**Mean**	75	82.7	87.3	89.8	90.5	87.6
**SD**	**23.93**	**21.04**	**18.23**	**15.4**	**15.39**	**16.39**
	**POST250**	**POST500**	**POST1000**	**POST2000**	**POST4000**	**POSTPTA**
**Mean**	56.8	58.3	66.4	70.7	74.7	67.5
**SD**	**29.67**	**27.68**	**23.76**	**20.92**	**20.55**	**21.4**
	**250GAIN**	**500GAIN**	**1000GAIN**	**2000GAIN**	**4000GAIN**	
**Mean**	18.2	24.3	20.9	19.2	15.8	
**SD**	16	15.6	14.7	11.5	12.8	
**t test**	7.85E-16	6.13E-18	1.22E-22	3.41E-28	1.78E-28	
**p value**	*p* < 0.05	*p* < 0.05	*p* < 0.05	*p* < 0.05	*p* < 0.05	

PTA = Pure Tone Average; PRE = Pre-treatment; POST = Post-treatment; SD = Standard Deviation; dB = Decibel; GAIN = Hearing gain (Pre-treatment– Post-treatment); n = Number of ears; p = p-value; t-test = Paired sample t-test

Note: PTA was calculated using the average thresholds at 500 Hz, 1000 Hz, 2000 Hz, and 4000 Hz

All p-values were statistically significant (*p* < 0.05) across all measured frequencies, indicating significant improvement in hearing thresholds after treatment

### Right ear audiometric parameters

Based on Siegel’s criteria, 21 out of 30 patients (70%) showed improvement (mild or partial), while 9 patients (30%) showed no improvement. Most patients initially had severe hearing loss (60%), and 40% had profound hearing loss. Treatment resulted in an average improvement of approximately 20.06 dB across frequencies. The pretreatment pure tone average (PTA) for the right ear was 87.58 ± 16.39 dB, which improved to 67.53 ± 21.40 dB after treatment. Specifically, 19 patients (63.33%) had mild improvement, 2 patients (6.67%) showed partial improvement, and nine patients (30%) had no improvement (Table 2).

As shown in Fig. 2A, correlation analysis revealed a moderate positive relationship between RF and RA (*r* = 0.56) and between ESR and HT (*r* = 0.43), suggesting a relevant clinical association between inflammatory markers and autoimmune diagnoses in BSHL patients. Additionally, age showed weak to moderate positive correlations with ANA (*r* = 0.21) and RA (*r* = 0.26), while female sex (coded as 1) was positively correlated with the presence of ANA (*r* = 0.32), SLE (*r* = 0.38), and RF (*r* = 0.29). As illustrated in Fig. 2B, autoimmune markers and diseases were more frequently observed in female patients (*n* = 20) compared to males (*n* = 10), particularly for ANA (7 vs. 1), RF (6 vs. 2), and SLE (7 vs. 2). These findings support the notion of a female predominance and an immune-mediated component in the pathogenesis of bilateral sudden hearing loss.

**Fig. 1:**
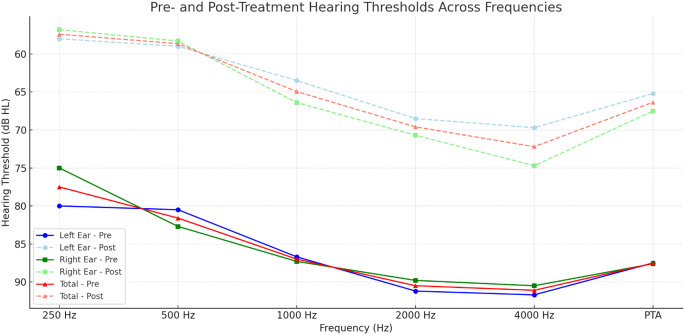
Frequency-Specific Pre- and Post-Treatment Hearing Thresholds in Left, Right, and Combined Ears of BSHL Patients

There is no significant difference between the left and right ears or between genders. However, all patients with no markers detected showed partial recovery (100%). In contrast, only 4 (13%) patients with positive markers had partial recovery (Table 2)(Fig. 1). Positive marker status impacts recovery negatively, as patients without markers demonstrated better outcomes compared to those with marker positivity.

**Fig. 2:**
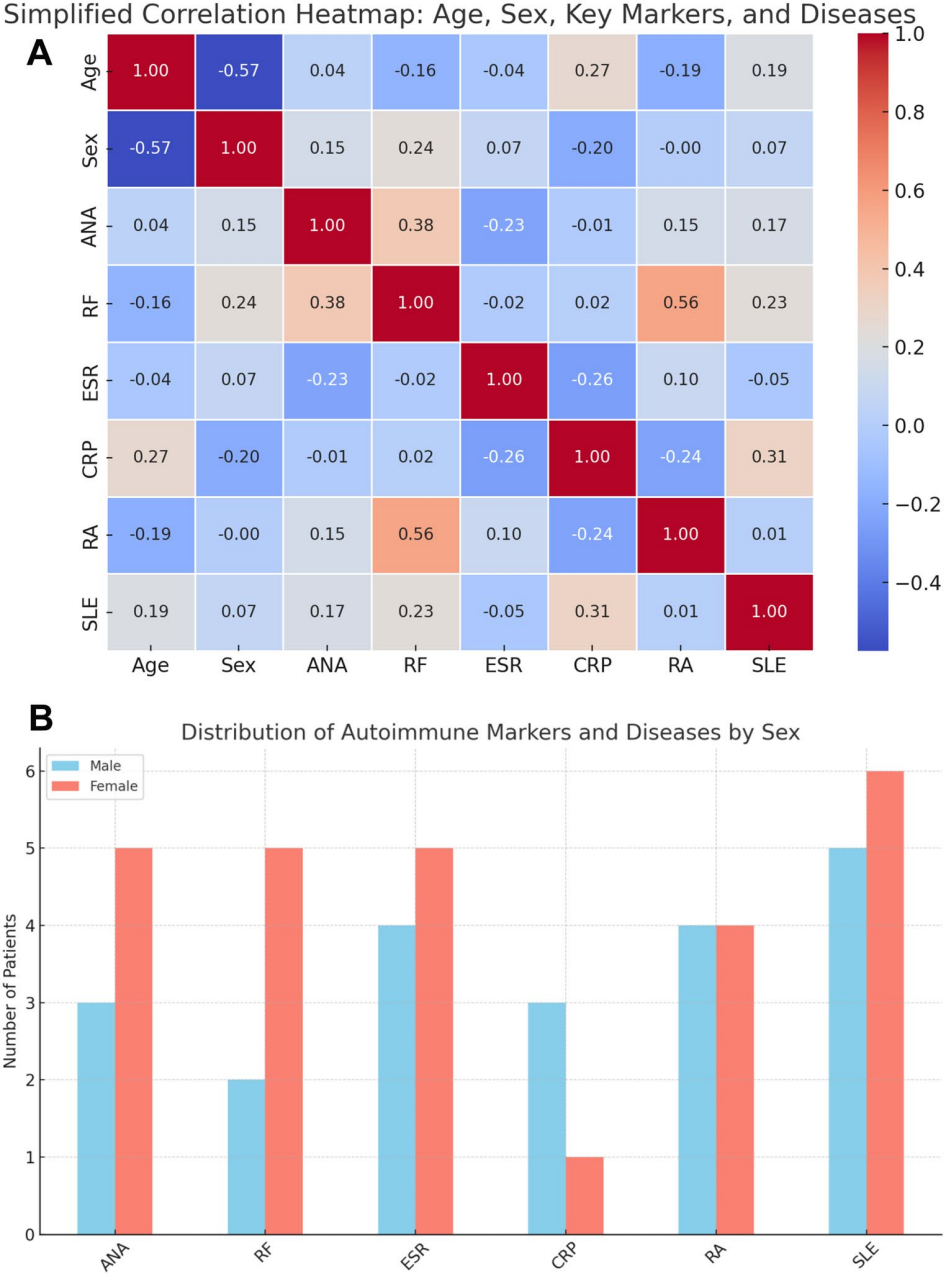
Distribution of Autoimmune Markers and Diseases in BSHL Patients. **A**. Heatmap of Autoimmune Markers and Autoimmune Diseases in BSHL Patients. **B**. Distribution of Autoimmune Markers and Autoimmune Diseases by Sex in BSHL Patients. *Note*: Combined visualization of autoimmune characteristics in BSHL patients. **A**. Correlation heatmap showing relationships between age, sex (Female = 1, Male = 0), follow-up duration, autoimmune markers, and diseases. **B**. Clustered bar chart illustrating sex-based distribution of autoimmune markers and diseases, with higher prevalence among female patients. ANA– Antinuclear Antibody, RF– Rheumatoid Factor, ESR– Erythrocyte Sedimentation Rate, CRP– C-Reactive Protein, ACA– Anticardiolipin Antibody, ANCA– Antineutrophil Cytoplasmic Antibody, AS– Ankylosing Spondylitis, HT– Hypothyroidism, RA– Rheumatoid Arthritis, SLE– Systemic Lupus Erythematosus, BSHL– Bilateral Sudden Hearing Loss

## Discussion

This study explores the role and importance of autoimmunity, particularly in bilateral sudden hearing loss (BSHL) cases, within the context of the existing literature. The etiology of SHL is complex and often multifactorial, but autoimmune mechanisms are increasingly recognized as a significant contributing factor. Although many factors are thought to play a role in the etiopathogenesis of sudden hearing loss, such as iron deficiency, vitamin D deficiency, genetics, and comorbid diseases, the most widely accepted theory is the autoimmune theory [9–13]. Numerous studies have highlighted the potential autoimmune origin of SHL, especially in bilateral cases, where the association with autoimmunity appears to be more robust [10, 13, 14]. In bilateral SHL, the pathophysiological processes driven by autoimmune responses seem more pronounced. This suggests that bilateral SHL represents a distinct disease process compared to unilateral SHL, potentially requiring different therapeutic approaches [11–13]. Our study supports this notion by identifying autoimmune and inflammatory markers in bilateral SHL patients and evaluating their role in diagnosis and treatment.

Yehudai et al. [14] highlighted that autoimmune sensorineural hearing loss (ASNHL) is associated with bilateral rapidly progressive hearing loss involving autoantibodies and autoreactive T cells. They also suggested that sudden hearing loss could be linked to antiphospholipid syndrome (APS), emphasizing the need for specific diagnostic tools to confirm autoimmune origins. García Berrocal et al. [15] further supported the recognition of immune-mediated SSNHL as a distinct entity, proposing that some patients may benefit from immunosuppressive treatment. Our study aligns with these findings, demonstrating elevated autoimmune markers in many patients with SSNHL.

Sudden sensorineural hearing loss (SSNHL) has been linked to autoimmune diseases, with markers indicating immune responses that may damage inner ear structures. Studies show that type II-IV allergic reactions involving markers like anti-heat shock protein 70 (HSP-70), and anti-type II collagen may lead to immune-mediated hearing loss. Biomarkers such as C-reactive protein (CRP), erythrocyte sedimentation rate (ESR), and specific antibodies can help detect autoimmune activity, though further research is needed to refine diagnostic accuracy [4, 16–19].

Li et al. [4] discussed autoimmune mechanisms in SSNHL, focusing on type II-IV allergic reactions, where immune responses targeting antigens like HSP-70 and collagen type II cause damage. This aligns with our use of inflammatory markers like CRP, ESR, and antibodies in identifying SSNHL’s autoimmune basis. Our study’s detection of these markers supports the idea that SSNHL may be autoimmune in nature for certain patients.

Choi et al. [17] linked psoriasis, an immune-mediated disease, and the study by Gomaa et al. [19] shows a higher risk of SSNHL, particularly in men, highlighting systemic inflammation’s role. This supports our study’s exploration of inflammatory markers like ANA, ANCA, and ESR in autoimmune comorbidities associated with SSNHL.

Incorporating the insights from Li, Choi, and Gomaa et al.into our research, we emphasize that immune dysregulation may play a crucial role in SSNHL development. Evaluating systemic biomarkers such as CRP, ESR, ANA, and RF can help identify underlying autoimmune activity that may not yet present with overt clinical symptoms. Similarly, assessing local biomarkers or inner ear-specific antibodies may reveal early immune-mediated damage, allowing for prompt initiation of immunomodulatory treatment before irreversible hearing loss occurs.

In the study by Oh et al. [20], the authors analyzed 324 patients with sudden sensorineural hearing loss (SSNHL), classifying them into bilateral (bi-SSNHL) and unilateral (uni-SSNHL) groups. They found that bi-SSNHL had a lower incidence (4.9%) and was associated with older age, diabetes mellitus, and lipid abnormalities. Additionally, the hearing recovery rate was significantly lower in the bi-SSNHL group, with only 37.5% of ears showing recovery compared to 56.5% in uni-SSNHL patients. This finding is consistent with our study, where patients with bilateral hearing loss also demonstrated a lower recovery percentage than those with unilateral loss, reinforcing that systemic factors, such as metabolic and inflammatory markers, may contribute to poorer prognosis in bilateral cases.

One limitation of our study is the small sample size, primarily due to the rarity of bilateral sudden hearing loss (BSHL). Establishing two distinct groups based on autoimmune marker positivity and negativity, is challenging. BSHL represents a very rare clinical condition, limiting the availability of cases for extensive analysis. However, the study’s strength lies in its focus on the autoimmune mechanisms in BSHL, which are not widely explored in the current literature. Despite the small sample size, the study provides valuable insights into the role of inflammatory and autoimmune markers in the prognosis of BSHL.

## Conclusion

Our study emphasizes the potential role of autoimmune and inflammatory mechanisms in bilateral sudden hearing loss (BSHL), as reflected by the presence of marker positivity in a significant number of patients. Although all patients received corticosteroid therapy, the overall recovery—particularly in those with positive markers—was limited, suggesting that corticosteroids alone may be insufficient in such cases. These findings highlight the need to consider alternative or adjunctive immunosuppressive strategies in marker-positive patients. Given the rarity of BSHL and the limited sample size, a multicenter study with a larger cohort is necessary to validate our results and further explore the optimal treatment approaches for autoimmune-related BSHL.

## Data Availability

The datasets used and/or analysed during the current study are available from the corresponding author on reasonable request.
